# Acute Pancreatitis Induced by Polatuzumab-Vedotin-Piiq in Combination With Bendamustine and Rituximab for Diffuse Large B-Cell Lymphoma

**DOI:** 10.7759/cureus.10299

**Published:** 2020-09-07

**Authors:** Kara Anderson, Mena Shehata, Davinder Singh, Mohammed Al-Ourani

**Affiliations:** 1 Internal Medicine, Marshall University Joan C. Edwards School of Medicine, Huntington, USA; 2 Pulmonary and Critical Care, Marshall University Joan C. Edwards School of Medicine, Huntington, USA; 3 Pulmonology and Critical Care, Marshall University Joan C. Edwards School of Medicine, Huntington, USA

**Keywords:** diffuse large b cell lymphoma, acute pancreatitis, polatuzumab vedotin-piiq, bendamustine, rituximab

## Abstract

While alcohol and gallstones have been considered the most common causes of pancreatitis, we investigate two uncommon etiologies such as drug-induced and viral-induced. Pancreatitis, an inflammatory process, can lead to many complicated outcomes such as acute respiratory failure, sepsis, and death. Examining drug-induced pancreatitis poses a large challenge for physicians as majority of the data is extrapolated from case reports. This is made even more difficult for patients who are on large chemotherapeutic regimens which include multitude of drugs and various side effects. Besides patient medication regimens, other etiologies of pancreatitis must be ruled out such as viruses. We examine a case of a 48-year-old female undergoing treatment for diffuse large B-cell lymphoma with various chemotherapeutic agents and positive cultures for varicella zoster virus (VZV) presenting with diffuse epigastric abdominal pain.

## Introduction

Pancreatitis is an inflammatory process that can lead to many sequalae including: sepsis, acute respiratory failure, and death [1]. Presentation involves epigastric pain, nausea, and vomiting. Serum lipase is diagnostic and imaging can confirm pathological changes [2]. While several reports focus on common etiologies such as condition of the patient, exposure, and genetic predisposition, two causes of pancreatitis that are not very common are drug-induced and virus-induced [1]. However, some patients are at a unique predisposition for this, namely immunocompromised individuals undergoing chemotherapy as they are exposed to both toxic medication and frequently have recurrence of latent infections [3]. Many chemotherapeutic agents have damaging effects on the pancreas. Furthermore, the immunosuppressed state can reactivate latent viruses, such as varicella, that can also induce pancreatic cell damage [3]. Here, we detail a case fatality due to acute respiratory distress syndrome secondary to acute pancreatitis caused by chemotherapeutic regimen: polatuzumab-vedotin-piiq (POLIVY), bendamustine, and rituximab for diffuse large B-cell lymphoma (DLBCL).

## Case presentation

A 48-year-old female with a history of follicular lymphoma transformed to DLBCL, hypertension, hyperlipidemia, and type 2 diabetes mellitus presented to her local ER with diffuse epigastric pain. Based on the symptoms and laboratory workup, her presentation was suggestive of pancreatitis. Ultrasound of the right upper quadrant failed to reveal any common bile duct dilatation or intraductal stone. CT of abdomen and pelvis showed uncomplicated pancreatitis with surgically absent gall bladder. She was diagnosed with acute pancreatitis. The patient’s social history was nonsignificant for alcohol use and trauma. Fluid resuscitation was implemented with initial improvement. She was discharged the next day but subsequently presented to our ED with continued pain that had increased in severity and duration, as well as, nausea and vomiting. On admission, the patient was in acute distress and was started on intravenous fluids (IVF) hydration and antibiotics (vancomycin and piperacillin tazobactam) due to pancytopenia and concerns for pneumonia. She had undergone a third cycle of POLIVY, bendamustine, and rituximab chemotherapy eight days before her initial presentation for epigastric pain. Note the patient had previously undergone treatment with bendamustine-rituximab, rituximab-cyclophosphamide-doxorubicin-vincristine-prednisone (RCHOP), rituximab-gemcitabine-dexamethasone-cisplatin (RGDC), rituximab-ifosfamide-carboplatin-etopaside (RICE), chimeric antigen receptor T cell (CAR-T), and obimutuzumab. However, the patient did not respond to the treatment and was switched to POLIVY, bendamustine, and rituximab chemotherapy. 

**Figure 1:**
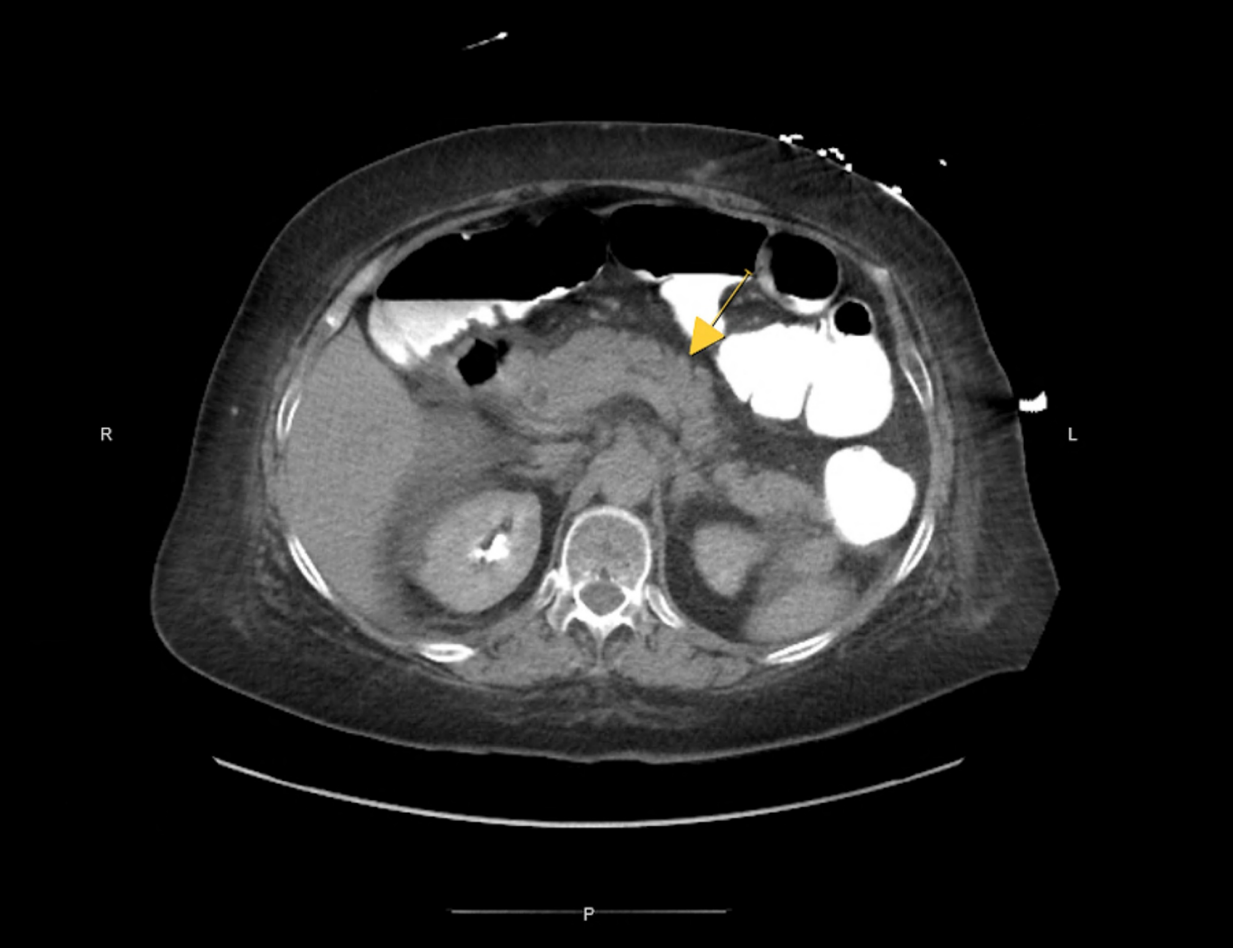
Pancreatitis without peripancreatic fluid collection or mass.

The patient on admission had the following laboratory findings: respiration 18 breaths/min, blood pressure 147/102 mmHg, heart rate 116 beats/min, and temperature oral 98.5 degree Fahrenheit. Physical exam was benign except for mild tenderness in the epigastric region and tachycardia. Lab work revealed white blood cell count 1.4 k/cmm (ref.: 4.5-10 k/cmm), hemoglobin 10.6 g/dL (ref.: 12-16 g/dL), hematocrit 30.2% (ref.: 35%-47%), platelets 38 k/cmm (ref.: 150-440 k/cmm), glucose 203 mg/dL (ref.: 74-100 mg/dL), aspartate transaminase (AST) 440 (ref.: 15-37), alanine transaminase (ALT) 248 (ref.: 12-78), bilirubin total 5.9 (ref.: 0.2-1.0), lipase 11,328 (ref.: 73-393), triglycerides 263 mg/dL (ref.: 10-150), and lactate dehydrogenase (LDH) was elevated to 508 unit/L (ref.: 140-280 units/L). Repeat CT showed persistence of the previously noted pancreatitis with no organized peripancreatic fluid collection or mass (Figure 1). Patient notable medications on admission included cetirizine, furosemide, glipizide, humalog, metoprolol, omeprazole, and ranitidine. 

The patient was admitted to the floor for management of ongoing pancreatitis. Due to increasing respiratory distress, she was put on continued positive airway pressure ventilation (CPAP) and moved to the ICU. On physical exam there, diffuse crackles were appreciated throughout all lung fields and she was tachycardic with an irregularly irregular rhythm. The patient developed atrial fibrillation with rapid ventricular rhythm and was treated with cardizem drip. She was also given filgastram, packed red blood cells, and platelet transfusions as needed for pancytopenia that was likely due to previous chemotherapy. On the seventh day of her stay, a vesicular rash was noted on the left side of her abdomen in a dermatomal distribution extending to the midline and back. She tested positive for varicella zoster virus (VZV) and was treated with IV acyclovir. In the ICU, she developed acute respiratory distress syndrome (ARDS) requiring mechanical ventilation with sedation achieved using lorazepam and hemodynamic stability achieved using norepinephrine. This persisted and was complicated by multiorgan system failure. Eleven days after admission, the family decided for comfort care.

## Discussion

This patient’s presentation offers a unique consideration for the etiology of acute pancreatitis. Based on her presentation and condition, the two likely sources of her pancreatitis were her recent chemotherapy treatment and varicella infection. This patient had follicular lymphoma that transformed to DLBCL that was refractory to multiple therapies. The patient had previously undergone bendamustine-rituximab, RCHOP, RGDC, RICE, CAR-T, and obimutuzumab treatment. She was started on a regimen that was based on the National Comprehensive Cancer Network Guidelines for relapse or refractory DLBCL. It included a combination of rituximab, bendamustine, and POLIVY. She was started on the treatment plan four months before the onset of her pancreatitis with the last cycle administered eight days before initial presentation.

Other common causes of pancreatitis include gallstones, alcohol abuse, hypertriglyceridemia, medications, and trauma [1]. Alcohol, gallstones, and trauma were ruled out based on that patient’s history. She was status post cholecystectomy which was confirmed on admitting abdominal ultrasound and abdominal CT, ruling out gallbladder stones or disease as an underlying etiology. She also did not consume alcohol and had no recent trauma. Triglycerides on admission were 263 mg/dL (ref.: 10-150); however, elevated triglycerides are generally not a risk factor for pancreatitis unless they are above 500 mg/dL [2]. Furthermore, elevated triglycerides can be a sign of worsening pancreatitis due to visceral fat necrosis and inflammation rather than the inciting cause [4]. On admission, the patient was on other medications to manage her chronic conditions including hypertension, diabetes, chronic pain, and gastroesophageal reflux disease (GERD). These medications included: cetirizine, furosemide, glipizide, humalog, metoprolol, omeprazole, and ranitidine. Pancreatitis is not a documented side effect for most of these medications as per the literature review [5-9].

There have been some case studies that showed isolated pancreatitis with acute use of these medications. One case report suggested furosemide as the cause of pancreatitis in individuals with underlying atherosclerosis due to poor perfusion [10]. The patient in this case had been put on furosemide for lower limb swelling due to complications of atherosclerotic disease and heart failure. The pancreatitis resolved with discontinuation of the medication and fluid resuscitation. Our patient had been taking furosemide chronically, making this a less likely source. While ranitidine has been documented to cause recurrent pancreatitis in limited studies our patient had been chronically using ranitidine with no adverse events documented. Finally, beta blockers were cited as the source of pancreatitis in a patient with an acute myocardial infarction [11]. The authors concluded that the beta blockade led to significant increases in triglycerides and subsequent pancreatitis. This patient had serum triglyceride of 4956 mg/dL. As stated, our patient’s triglyceride on admission, while elevated, was only 263 mg/dL, making this an unlikely cause as well [11]. 

Chemotherapy-induced pancreatitis is rarely reported. Although acute pancreatitis is sometimes associated with l-asparaginase based therapies there are still several clinical trials for other chemotherapeutic agents where patients end therapy due to development of pancreatitis [12]. In one documented case a patient with testicular cancer being treated with cisplatin, bleomycin, and vinblastine developed signs of acute pancreatitis seven days after the end of his first and second cycle of treatment with no other known risk factors [12]. The authors suggest there may be a correlation between metastatic disease and drug infiltration into the pancreas [12]. They also point out the varying evidence of chemotherapy and pancreatic disease. Further discussion here will highlight those studies based on the chemotherapeutic regimen of our patient: bendamustine, rituximab, and POLIVY.

Bendamustine is an alkylating agent that induces cell death through intra-strand cross links between DNA. It is commonly used in combination with rituximab, a monoclonal anti-CD20 antibody, for the treatment of several hematological conditions including relapsed/refractory DLBCL [13]. Several studies observing treatment toxicity report the most common effects being myelosuppression and nausea/vomiting [13]. The drug facts related to bendamustine do not endorse acute pancreatitis as an adverse event but do report increases in liver enzymes and creatine. In a retrospective study of 1095 cycles where bendamustine was a component of therapy, there were two episodes of pancreatitis and reactivation of a VZV infection occurring in seven (3%) patients [14]. In another study of a bendamustine/rituximab combination therapy with ibrutinib, acute pancreatitis was a complication in one of their 48 patients (2%) [15]. In a clinical review on 12,448 adverse events reported to the FDA for rituximab, there were no cases involving pancreatitis [16]. Commonly reported effects usually include neutropenia, pneumonia, and anemia [16]. Among the gastrointestinal symptoms reported, the most common events were bowel obstruction and perforations with the average onset of symptoms being six days after treatment [16]. There are no case reports indicating bendamustine or rituximab as the cause of acute pancreatitis, only clinical trial report as an adverse event.

Polatuzumab-vedotin-piiq is an anti-CD79B antibody used in refractory B-cell non-Hodgkin's lymphoma (NHL), such as this patient, where other modalities have failed. The drug has activity against specific targets on a component of mature B-cells as well microtubule disrupting capabilities due to its conjugation to a microtubule-disrupting agent, monomethyl auristatin E (MMAE) [17]. In a phase 2 clinical trial determining safety and clinical activity of the drug, 95 patients were enrolled in different arms based on the underlying diagnosis and drug therapy combination. The most common adverse reactions for the arm of the study involving patients with NHL included: neutropenia, anemia, and peripheral neuropathy [17]. There was no report of pancreatitis as an adverse event in patients after 21 days of treatment [17]. Similarly, in a study assessing 40 patients using the combination of POLIVY with bendamustine and rituximab to treat DLBCL, significant adverse drug reaction included diarrhea, infections, fatigue, pancytopenia, and peripheral neuropathy [18]. Pancreatitis was not a complication of any patients in their cohort with between three and five cycles of therapy completed [18]. In 66 patients with DLBCL treated with POLIVY in combination with R-CHOP therapy, there were two treatment-related deaths associated with atrial fibrillation and sepsis of unknown etiology [19]. In all studies reviewed involving POLIVY therapy, there were no case fatalities reported due to pancreatitis-related complications. This indicates that this case may be the first report of this adverse event for this chemotherapeutic agent.

CD79B, the target of POLIVY, is found on mature B-cells making it an ideal target for the treatment of NHL [17]. This antigen has also been shown to be elevated in both chronic pancreatitis and pancreatic cancer, suggesting it may have a site-specific elevation in the pancreas [18-19]. They are derived from inflammatory cells that may have an origin in pancreatic parenchyma [18]. Although there have not been any studies on the correlation between CD79B targeted treatment and direct pancreatic damage, CD79 receptors native to the pancreas may be affected during treatment with this therapy, inducing inflammation and leading to acute pancreatitis.

The other component of our patient’s presentation to consider was her infection with varicella. Herpes zoster, caused by reactivation of varicella virus, is a very rare cause of acute pancreatitis and is often correlated with immune suppression [3]. Our patient did not develop a rash until several days into admission, suggesting that her herpes simplex virus (HSV) infection more likely exacerbated her pancreatitis, rather than caused it in a synergistic fashion. In a recent case report about a newly diagnosed HIV patient, their initial presentation was a vesicular rash proceeding pancreatitis. Treatment with fluid resuscitation and acyclovir resolved both symptoms [3]. Like our patient, the immunocompromised nature of the patient in this report led to the varicella infection. However, signs of a varicella infection were presented prior to the onset of their abdominal symptoms and subsequent pancreatitis diagnosis. There have been some reports suggesting the vesicular formation indicative of an underlying varicella infection could be delayed or absent in this patient population [20]. However, with our case, antibody testing for varicella was negative at admission of our patient and polymerase chain reaction (PCR) was only possible after the eruption of a rash. Furthermore, the patient had a localized rather than disseminated zoster infection as it had affected only one dermatomal distribution. This makes it more likely that the cause of our patient’s acute pancreatitis was more likely due to her recent chemotherapy, rather than an infection. Regardless, immunocompromised patients with herpes zoster positive serology should prompt suspicion for visceral seeding [3, 20]. Patients with any history of varicella and are undergoing treatment that could put them in an immunocompromised state should receive valacyclovir prophylaxis [20].

## Conclusions

Our patient’s acute pancreatitis was secondary to her recent chemotherapy. There is some evidence that POLIVY, bendamustine, and rituximab can induce acute pancreatitis; however, there are neither case reports that specifically detail the course of this infection. Nor do any reports have fatality as a complication for these treatments secondary to pancreatitis. This is an important adverse event to report, especially for the treatment of patients with this regimen that may have other risk factors for pancreatitis including: hypertriglyceridemia, cholestatic disease, or take other medication known to induce pancreatic damage. This patient also had a superimposed varicella infection which could have exacerbated her clinical course of pancreatitis in a synergistic fashion. This adds to the discussion of the need for antiviral prophylaxis for immunocompromised patients who have a history of varicella infection.
